# Supplementary material for: Rapid reconstitution and kinase-controlled regulation of algal pyrenoid condensates in engineered Nicotiana benthamiana
Source: bioRxiv. 2026 Jul 21:2026.07.20.739623. Preprint. [Version 1] doi: 10.64898/2026.07.20.739623 (PMC13419457; doi:10.64898/2026.07.20.739623)
Supplement: Supplementary file 1 [file NIHPP2026.07.20.739623V1-supplement-1.pdf]

bioRxiv preprint doi: https://doi.org/10.64898/2026.07.20.739623; this version posted July 21, 2026. The copyright holder for this preprint (which was not certified by peer review) is the author/funder, who has granted bioRxiv a license to display the preprint in perpetuity. It is made available under a CC-BY 4.0 International license.

Supplementary Figure 1

a

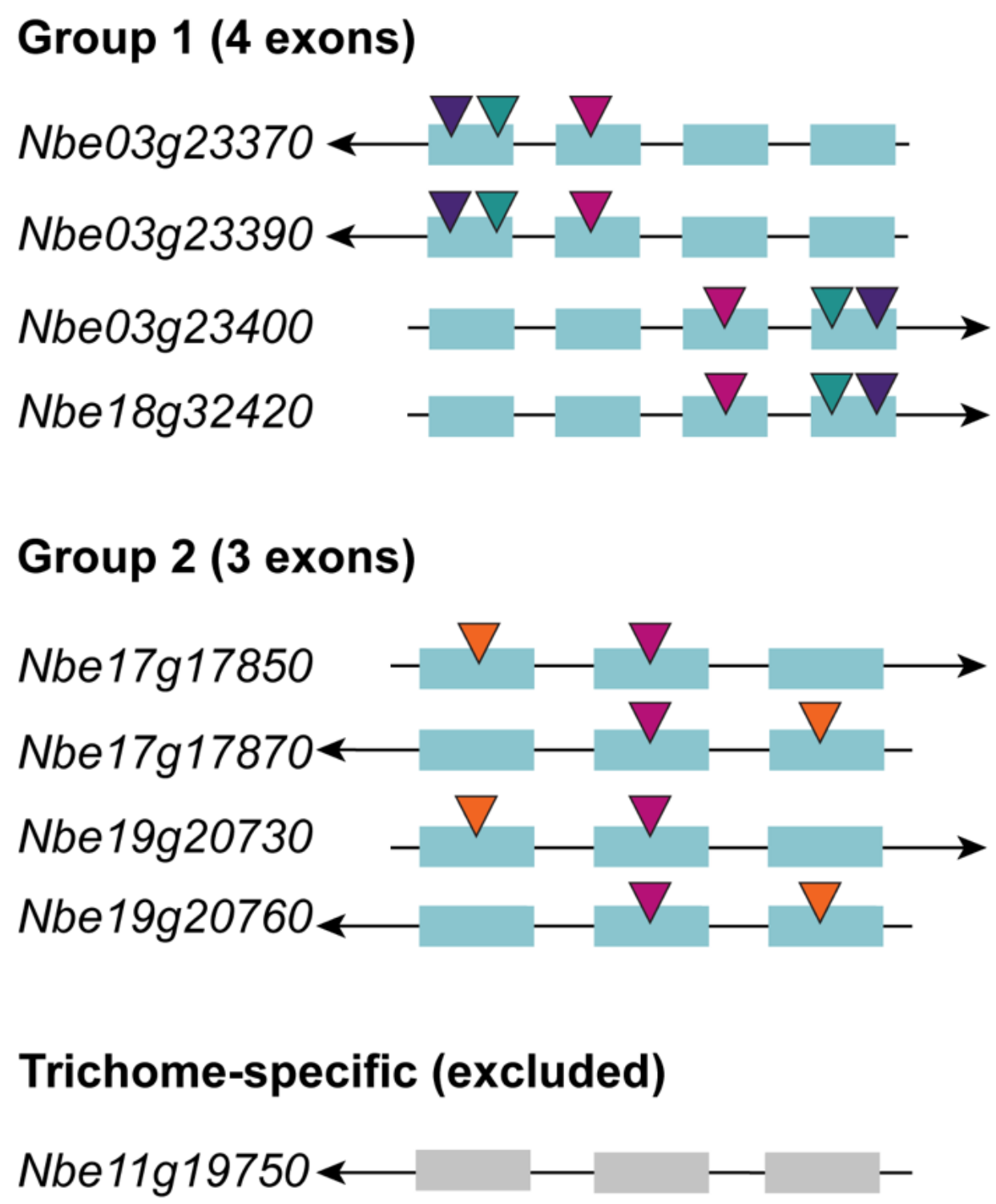

b

Guide	Sequence	PAM	Target group
▼	AAATGGATGGGTTCCTTGCT	TGG	All 8 loci (exon 2)
▼	CAATGTTGCTCAAGCTAACA	TGG	4-exon group (exon 1)
▼	CGGAAGAGTGCAATGCATGC	AGG	4-exon group (exon 1)
▼	TGTGGAAGTTGCCCATGTTC	GGG	3-exon group (exon 3)

Supplementary Figure 1. Gene structure and sgRNA target sites of photosynthetic *NbRBCS* loci in *N. benthamiana*.

(a) Gene models of the eight photosynthetic *NbRBCS* loci identified in the *N. benthamiana* genome assembly. Loci are grouped by exon number: Group I (4 exons) and Group II (3 exons). Arrows indicate transcription orientation. Colored triangles indicate sgRNA target positions. One trichome-specific locus (Nbe11g19750) is shown in gray and was excluded from targeting.

(b) Sequences of the four sgRNAs used for *NbRBCS* disruption, with corresponding PAM sequences and target groups.

bioRxiv preprint doi: https://doi.org/10.64898/2026.07.20.739623; this version posted July 21, 2026. The copyright holder for this preprint (which was not certified by peer review) is the author/funder, who has granted bioRxiv a license to display the preprint in perpetuity. It is made available under a CC-BY 4.0 International license.

Supplementary Figure 2

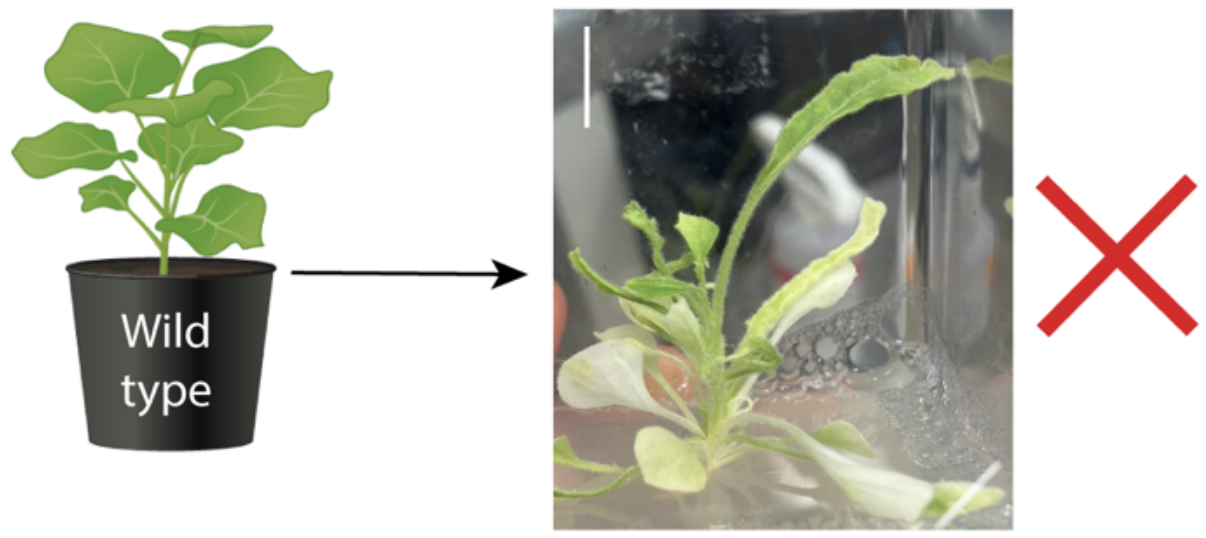

Supplementary Figure 2. Stable transformation with a Cas9/sgRNA construct targeting *NbRBCS* genes does not yield viable *Nbrbcs* plants.
Representative T0 regenerant following stable transformation with a Cas9/sgRNA construct targeting *NbRBCS* genes. T0 regenerants failed to root on sucrose-supplemented Murashige and Skoog (MS) medium and died before completing a full life cycle. Complementation with *CrRBCS2* was unsuccessful. Scale bar, 1 cm.

bioRxiv preprint doi: https://doi.org/10.64898/2026.07.20.739623; this version posted July 21, 2026. The copyright holder for this preprint (which was not certified by peer review) is the author/funder, who has granted bioRxiv a license to display the preprint in perpetuity. It is made available under a CC-BY 4.0 International license.

Supplementary Figure 3

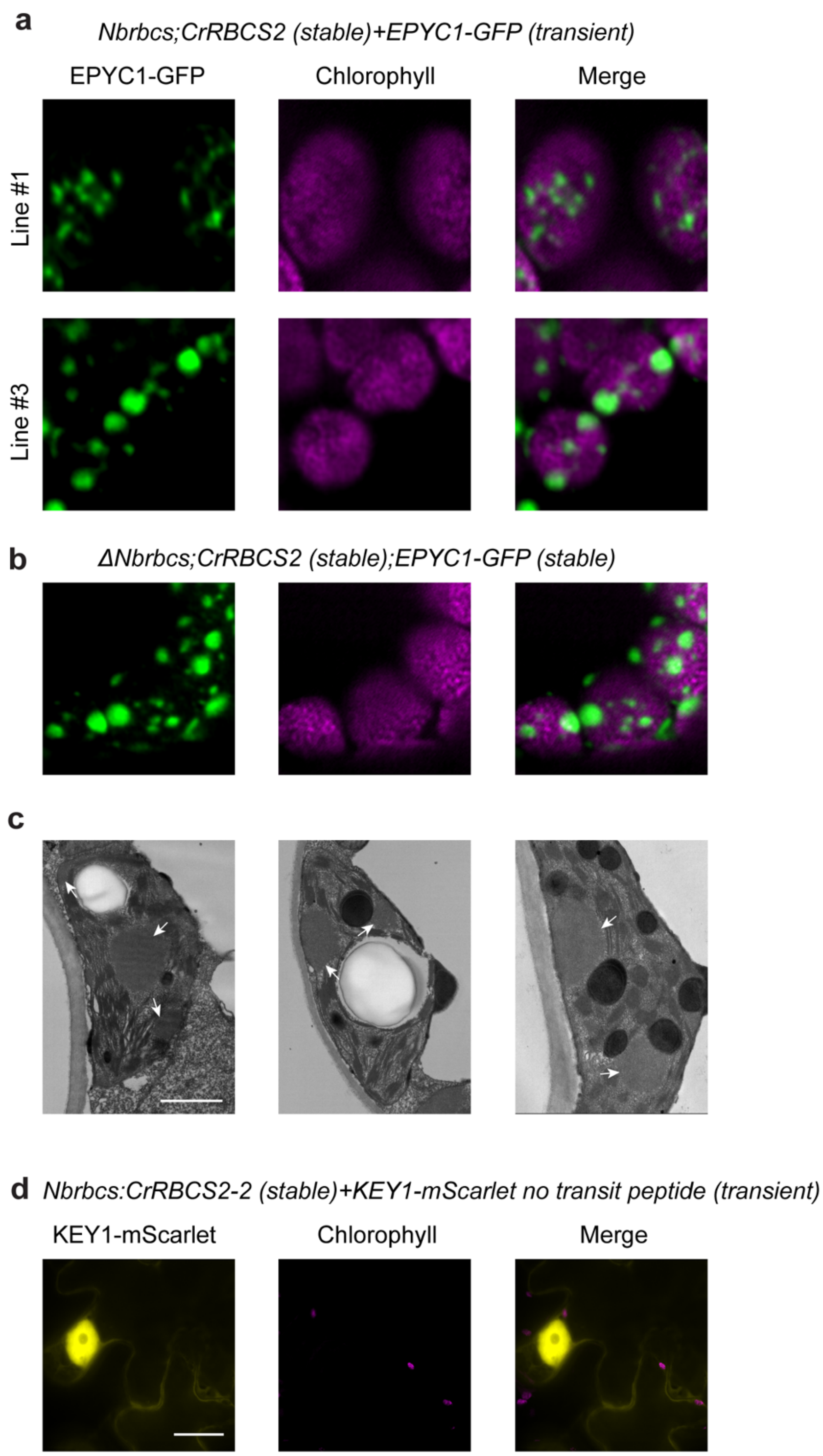

bioRxiv preprint doi: https://doi.org/10.64898/2026.07.20.739623; this version posted July 21, 2026. The copyright holder for this preprint (which was not certified by peer review) is the author/funder, who has granted bioRxiv a license to display the preprint in perpetuity. It is made available under a CC-BY 4.0 International license.

Supplementary Figure 3. Additional characterization of EPYC1-GFP condensates and KEY1-mScarlet localization in *Nbrbcs;CrRBCS2* chloroplasts.

(a) Confocal images of EPYC1-GFP localization in *Nbrbcs;CrRBCS2* Line 1 and Line 3 following transient expression. Chlorophyll autofluorescence is shown in magenta. Scale bar, 5 µm.

(b) Confocal images of EPYC1-GFP localization in *Nbrbcs;CrRBCS2* plants following stable expression. Chlorophyll autofluorescence is shown in magenta. Scale bar, 5 µm.

(c) Transmission electron microscopy of *Nbrbcs;CrRBCS2;EPYC1-GFP* chloroplasts. Arrows indicate electron-dense structures. Scale bars, 1 µm (overview) and 500 nm (insets).

(d) Confocal images of KEY1-mScarlet expressed without a chloroplast transit peptide in *Nbrbcs;CrRBCS2* plants. Chlorophyll autofluorescence is shown in magenta. Scale bar, 5 µm.

bioRxiv preprint doi: https://doi.org/10.64898/2026.07.20.739623; this version posted July 21, 2026. The copyright holder for this preprint (which was not certified by peer review) is the author/funder, who has granted bioRxiv a license to display the preprint in perpetuity. It is made available under a CC-BY 4.0 International license.

Supplementary Table 1. Photosynthetic parameters of *Nbrbcs;CrRBCS2* complemented lines and wild-type *N. benthamiana*.

Genotype	n	V_{cmax} mean ± SD (μmol CO_2 m^{-2} s^{-1})	J_{max} mean ± SD (μmol e^- m^{-2} s^{-1})	G_s at ambient (mol m^{-2} s^{-1})
Wild type	5	78.2 ± 6.3	100.10 ± 5.8	0.17 ± 0.03
Line 1	7	16.9 ± 2.8	44.5 ± 7.2	0.18 ± 0.05
Line 2	7	26.1 ± 4.9	59.8 ± 11.3	0.25 ± 0.04
Line 3	7	22.7 ± 4.7	50.8 ± 11.08	0.16 ± 0.05

V_{cmax}, maximum rate of Rubisco carboxylation; J_{max}, maximum rate of electron transport; G_s, stomatal conductance at ambient CO_2. Parameters estimated by fitting A/C_i curves to the Farquhar–von Caemmerer–Berry model (Farquhar et al., 1980) using kinetic constants from Bernacchi et al. (2001). Values are mean ± SD. G_s measured at ambient CO_2 (~400 μmol mol^{-1}).
